# Veterans Affairs FreshConnectProduceRx: a study protocol for a pragmatic quasi-experimental study assessing health, healthcare costs, and implementation processes of a produce prescription program in VA medical centers

**DOI:** 10.1186/s12889-025-23355-2

**Published:** 2025-07-03

**Authors:** Jorie M. Butler, Nipa Kamdar, Diana P. Brostow, Ronit Ridberg, Dariush Mozaffarian, Diana Johnson, Megan Bowman, Adam Shyevitch, Christine Going, Yue Zhang, Sara Napa, Joan Heusser, Steven Prater, Sandra Posada, Richard Nelson

**Affiliations:** 1 Department of Biomedical Informatics, University of Utah School of Medicine, Salt Lake City, UT USA; 2 Department of Internal Medicine, Division of Geriatrics, University of Utah School of Medicine, Salt Lake City, UT USA; 3 Salt Lake City VA Informatics Decision-Enhancement and Analytic Sciences (IDEAS) Center for Innovation, George E. Wahlen Veterans Affairs Medical Center, Salt Lake City, UT USA; 4 Geriatrics Education and Clinical Center (GRECC) George E. Wahlen Veterans Affairs Medical Center, Salt Lake City, UT USA; 5 Department of Medicine, A Center for Innovations in Quality, Effectiveness, and Safety, Michael E. DeBakey Veterans Affairs Medical Center, Houston, TX USA; 6 Baylor College of Medicine, Houston, TX USA; 7 Veterans Health Administration Rocky Mountain Mental Illness Research Education and Clinical Center, Rocky Mountain Regional Veterans Affairs Medical Center, Aurora, CO USA; 8 Department of Physical Medicine & Rehabilitation, University of Colorado Anschutz Medical Campus, Aurora, CO USA; 9 Department of Psychiatry, University of Colorado Anschutz Medical Campus, Aurora, CO USA; 10 Friedman School of Nutrition Science and Policy, Food Is Medicine Institute, Tufts University, Boston, MA USA; 11 The Rockefeller Foundation, New York, NY USA; 12 Nutrition and Food Services at George E. Wahlen Veterans Medical Center, Salt Lake City, UT USA; 13 About Fresh, Inc., Boston, MA USA; 14 Veterans Health Administration Office of the Assistant Deputy Undersecretary for Health for Clinical Operations, Washington, DC USA; 15 Department of Internal Medicine, Division of Epidemiology, University of Utah School of Medicine, Salt Lake City, UT USA; 16 Department of Medicine, Michael E. DeBakey Veterans Affairs Medical Center, Houston, TX USA

**Keywords:** Food insecurity, Food security, Nutrition security, Veterans, Pragmatic, Produce prescription, Quasi-experimental, Diabetes, Hypertension, Obesity

## Abstract

**Background:**

Food insecurity, poor nutrition, and diet-related diseases create major intersecting health challenges. The Veterans Health Administration (VHA) has identified food insecurity as a high-priority problem and established regular clinical screening. Veterans with identified food insecurity and diet-sensitive cardiometabolic health conditions will benefit from the successful implementation of effective Food is Medicine interventions.

**Methods:**

This pragmatic, quasi-experimental intervention study of effectiveness and implementation of a produce-prescription program is conducted in 2 VA hospital health systems in Salt Lake City, Utah, and Houston, Texas. Eligible Veterans have (a) a diet-sensitive cardiometabolic health condition (obesity, hypertension, and/or diabetes) identified in the electronic health record (EHR) by diagnostic codes (ICD-10) and/or lab values and (b) low-income identified by priority status in administrative data. Program enrollment is pragmatically integrated within the VA clinical care process of food security screening and service referrals. Eligible Veterans who screen positive for food insecurity during clinical care processes are referred to the intervention. The Veterans Affairs FreshConnect Produce Prescription (VA FCPRx) intervention program includes 12 months of a produce prescription allowance for purchasing fresh fruits or vegetables, provided as $100 monthly on a pre-paid card for use at local grocery stores. The program also includes culinary education through cooking courses provided by VA nutritionists or nutritional consults provided one-on-one by a VA dietitian. Process and outcome measures will be evaluated using the PRISM RE-AIM framework. Health outcomes related to diet-sensitive chronic conditions (e.g., HbA1c levels for patients with diabetes) and healthcare costs (e.g., outpatient costs) are assessed using EHR data. VA FCPRx participant outcomes are assessed in comparison to a group of similar Veterans using intention-to-treat analyses. Patient-reported outcomes, implementation strategies and outcomes, and staff and Veteran experience are assessed with a combination of surveys, focus groups, and program administrative data.

**Discussion:**

This pragmatic quasi-experimental intervention study will provide important new evidence about the impact of a produce prescription program for U.S. Veterans on health outcomes, healthcare costs, and patient-reported outcomes. The assessment of effectiveness and implementation processes and outcomes will inform the design and scaling of impactful, pragmatic, cost-effective programs for food insecure Veterans with diet-sensitive cardiometabolic conditions.

**Supplementary Information:**

The online version contains supplementary material available at 10.1186/s12889-025-23355-2.

## Background

Food insecurity is the lack of consistent, readily available, and safe food and affects approximately 17 million Americans, or 12.8% of the population [1, 2]. In addition to a sufficient quantity of food, ‘nutrition security’, or access to adequate quantities of food of appropriate nutritional value, is increasingly complementing the concept of food security, as it emphasizes dietary quality beyond caloric sufficiency [3, 4]. Food insecurity is a major threat to health and well-being [5–9]. The nutritional quality of the food is a crucial mediator of health outcomes. Food insecurity and poor nutrition are associated with an increased risk for multiple chronic diet-sensitive health conditions, including diabetes and related metabolic disturbances, obesity, hypertension, and cardiovascular disease [10]. A growing body of research has outlined the extent to which food or nutrition insecurity can impede diabetes management, [11–13] present as a barrier to treating obesity, [14, 15] and disproportionately influence cardiovascular outcomes. [16] Food insecurity is interlinked with other complex social needs [17, 18].

“Food is Medicine” or “Healthcare by Food” interventions to address food and nutrition insecurity, social needs, and health employ broad strategies to address complex challenges [19–22]. Food is Medicine interventions target specific diet-sensitive health conditions and social needs [21]. The goals of these interventions are to attenuate the impact of food insecurity, improve nutrition, help address economic social needs, and improve health and health inequities in at-risk populations [23]. Specific Food is Medicine intervention strategies include providing healthful food directly from healthcare organizations with curated food boxes or medically-tailored meals [24] or produce prescriptions to purchase fruits and vegetables [25]. These interventions have the potential to be cost-effective or even cost-saving for healthcare systems, given the far-reaching impacts of food insecurity and poor diet quality [26].

Thus far, produce prescriptions have been studied less often than other Food is Medicine strategies [27]. A systematic review and meta-analysis examining pre/post cardiometabolic outcomes in produce prescription recipients across 13 different studies demonstrated reductions in body mass index (BMI) and hemoglobin A1c (HbA1c) and improvements in eating behaviors, such as food and vegetable intake [28]. Results from randomized trials have demonstrated mixed findings for HbA1c impact in produce prescription participants with diabetes compared to controls [29]. The variability in these findings may relate to heterogeneity of produce prescription program designs, implementation approaches, and patient populations. Studies that include education components, such as provision of recipes and cooking courses (often referred to as culinary education) have a higher potential for health impact [19, 21, 28, 30]. A large review of studies that included a culinary education component demonstrated increases in fruit and vegetable intake, and suggested beneficial changes in HbA1c and BMI [31]. Of note, studies to date have generally been relatively small and many are 6 months or less in duration.

U.S. Veterans are vulnerable to experiencing food insecurity with prevalence estimates ranging from 6 to 22% [6–9]. Survey estimates for sub-populations of Veterans during the COVID-19 pandemic were even higher, above 30% [32, 33]. Poor diet can also exacerbate mental health conditions. The burden of chronic illness and mental health conditions can each affect income and earning potential, influence spending tradeoffs, and promote even poorer eating habits, creating a feedback loop of stress and poor health [34]. The Veterans Health Administration (VHA) began a large-scale program screening Veterans for food security during healthcare encounters in 2017 [35], providing an ideal avenue for identifying potential participants for a pragmatic study. This study is large, multi-site, focused uniquely on Veterans, conducted within the largest integrated healthcare system in the U.S., uses a grocery-focused produce prescription intervention, and incorporates Electronic Health Record (EHR) based outcomes. This combination of factors is being addressed for the first time in this study.

### Aims

The aims of this protocol are to describe the VA Fresh Connect Produce Prescription Program (VA FCPRx), a pragmatic, quasi-experimental study designed to assess the impact of a produce prescription program 12 months in duration on Veterans with food insecurity and specific diet-sensitive cardiometabolic conditions. In this protocol, we describe the effectiveness study and characterize implementation and process measures for VA FCPRx including program enrollment processes, participation, satisfaction, and implementation strategies.

## Methods

### Study design

Veterans experiencing or at risk for food insecurity, diagnosed with a cardiometabolic diet sensitive condition including hypertension, obesity, and/or diabetes, and a comparison group of similar Veterans will be enrolled in VA FCPRx to estimate the effect of produce prescription on clinical measures, healthcare utilization, and self-reported behavior and well-being.

Primary outcomes: Clinical measures, including body mass index (BMI), blood pressure, and metabolic control (HbA1c) derived from the VA EHR.

Secondary outcomes: Healthcare utilization, including primary care visits, pharmacy utilization, inpatient admissions, and ED visits. Veteran-reported health outcomes, including subjective ratings of health.

Exploratory outcomes: Veteran-reported consumption of fruits and vegetables, nutrition security, depression symptoms, and other well-being indices.

We will also assess implementation processes and outcomes.

### Setting

The study is conducted at VA Salt Lake City and VA Houston Medical Centers. These VA facilities were chosen based on prior pilot food security interventions successfully implemented at these sites.

### Program oversight and ethics review

Given the partnerships between the evaluation study team (centered at the University of Utah), the funder (The Rockefeller Foundation), the non-profit organization providing the produce incentives (About Fresh), and the VHA, there was a need for a governance structure to guide implementation activities and data agreements. An Innovation Cooperative Research and Development Agreement (CRADA) governing information sharing, and funds management was completed to enable program implementation, processes, and evaluation across multiple institutions. The University of Utah Institutional Review Board and Salt Lake City VA Research and Development Committee reviewed and approved all evaluation study procedures.

### Eligibility and screening

Eligible participants are identified using VA EHR data from among currently active patients defined by a visit to a participating VA medical center within the preceding 12 months. Inclusion criteria are:

1) An indicator in the VA EHR for economic hardship (Designated membership in VA benefits criteria priority groups 5 or 7, based on Veteran benefits. These groups indicate a Veteran who has low-income without a service-connected disability).

2) One or more diet-sensitive cardiometabolic condition a) hypertension, b) obesity, or c) type 2 diabetes or malnutrition related diabetes. Cardiometabolic condition is determined by diagnostic code within the VA EHR (International Classification of Disease, ICD-10) or by laboratory values. Table 1 shows the specific EHR ICD-10 codes and laboratory values. Consent is not required for this screening or identification process.

**Table 1:**
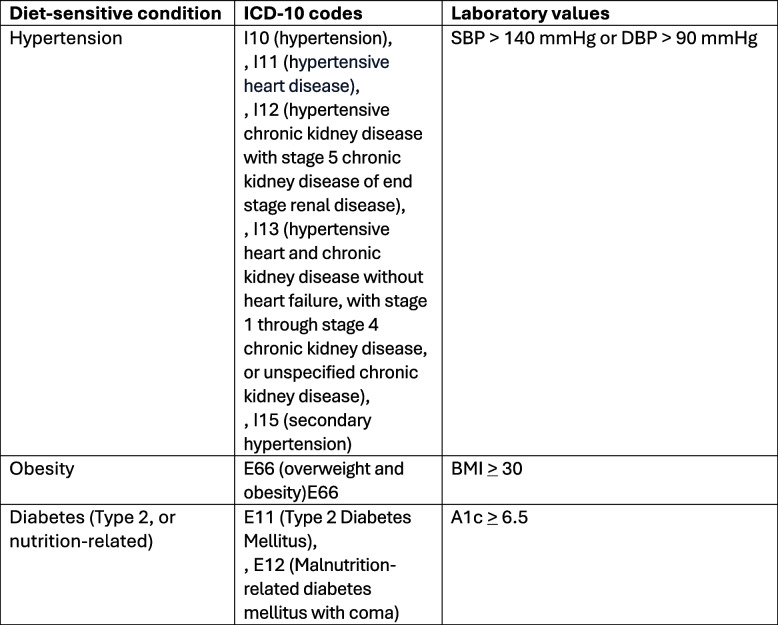
ICD-10 codes and laboratory values used to identify diet-sensitive conditions

### Intervention enrollment process (Screening)

Veterans who meet eligibility criteria are identified by a monthly VA EHR review by the evaluation team. Intervention enrollment will total at least 500 (250 per site). Enrollment will be closed once enrollment targets are reached. This was derived based on a power analysis performed on the primary outcomes coupled with practical considerations relating to the largest number of Veterans for whom the intervention and supportive health services would be available (see outcomes variables section for power analysis information). Of note, a recent systematic review of produce prescription programs included studies with adult sample sizes ranging from 21–243, thus, our planned sample of 500 is relatively large for the field to date [36]. Enrollment in the intervention group will continue until the planned sample size (*n* = 500) is reached.

**Fig. 1:**
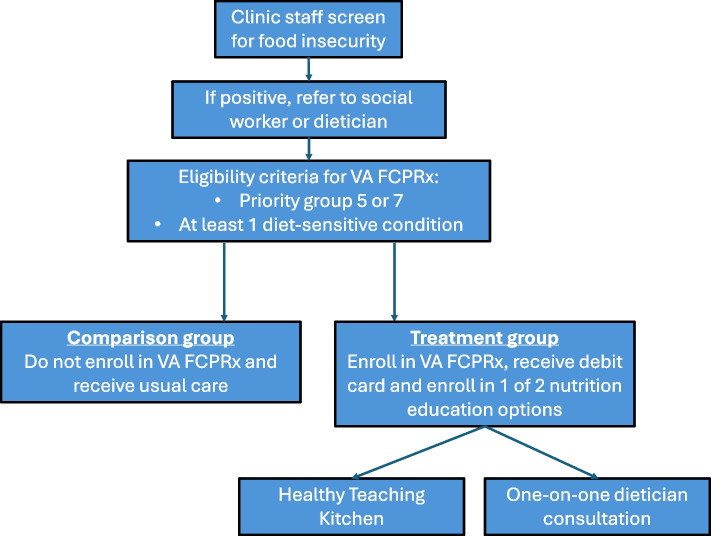
Flow chart outlining the process for the selection and exclusion of the studies following the PRISMA statement

A roster of eligible participants is made available on a secure Microsoft SharePoint site. This site is accessible only to the evaluation team and authorized nutritionists, dietitians, and social workers providing care to food-insecure Veterans. This screening approach supports the pragmatic enrollment process in this study. A Veteran who screens positive for food insecurity during clinical care is then referred to a social worker or dietitian. Social workers and dietitians at each study site are instructed to review the roster and offer FCPRx to Veterans who qualify (see Fig. 1). Veterans sign a release of information to permit information sharing with the Produce Prescription team. Figure 1 shows the study selection flow.

Social workers and dietitians subsequently enroll eligible and interested Veterans in VA FCPRx. The process of enrollment is supported by an EHR-integrated template developed for this study and available in the VA Computerized Patient Record System (CPRS) (see Additional File 1). CPRS is the operating system in use during the study period at both study sites [37–39]. The template supports service providers in each step of the enrollment process, including screening, information gathering, and obtaining the release of information. This informatics-based approach was chosen because it is scalable to other VA sites. In addition, this approach can be adapted to VA sites that have transitioned to another EHR system [40, 41].

### VA FCPRx program

#### Produce prescription benefit

The produce prescription benefit is provided by the non-profit organization About Fresh^© ^ [42]. Enrolled Veterans receive a pre-paid card with a monthly allowance of $100 for 12 months, which can be used at participating grocery stores to purchase fresh fruits and vegetables. Each month’s prescription benefit must be used during the month of issuance and does not “roll over” into subsequent months. Participating Veterans receive a combination of live phone and text support from About Fresh for any challenges with card usage. Participating grocery stores include large, national, and locally based stores throughout Texas, Utah, and surrounding states, such as Albertson’s, Kroger/Smith’s, and Wal-Mart [43].

#### Culinary education

Along with the monetary benefit of the program, participants will receive education designed to help them integrate the purchased produce into their diet, with a participant choice of: (1) a supportive nutrition class delivered in VA called Healthy Teaching Kitchen, offered virtually once per week for 4 weeks or (2) one-on-one consultation with a VA dietitian.

#### Comparison group participants

The comparison group for our study will consist of Veterans who meet eligibility criteria at study sites (e.g., screening and cardiometabolic conditions) but did not participate in the intervention. It is anticipated that this group will be enrolled after the intervention group has reached capacity. This comparison group was chosen based on likely demographic similarity and the study goal for a pragmatic rather than a randomized design focus on a program embedded in VA clinical care. There are multiple potential reasons Veterans might qualify for inclusion but not enroll in VAFCPRx, including not being offered enrollment by clinical staff during the pragmatic clinic enrollment process or because they decline to participate.

#### Implementation and effectiveness outcomes

The VA FCRPRx study metrics are based on the Practical Implementation Sustainability Model (PRISM) and related Reach, Effectiveness, Adoption, Implementation, and Maintenance (RE-AIM) framework to assess outcomes and implementation [44]. Comparisons of health and healthcare cost outcomes derived from VA EHR data, including BMI and pharmacy utilization. Implementation metrics were selected to inform scalable implementation at additional future VA sites and future sustainment of the program.

Specific metrics, measurement strategies, and analytic approach are outlined in Table 2.

**Table 2:**
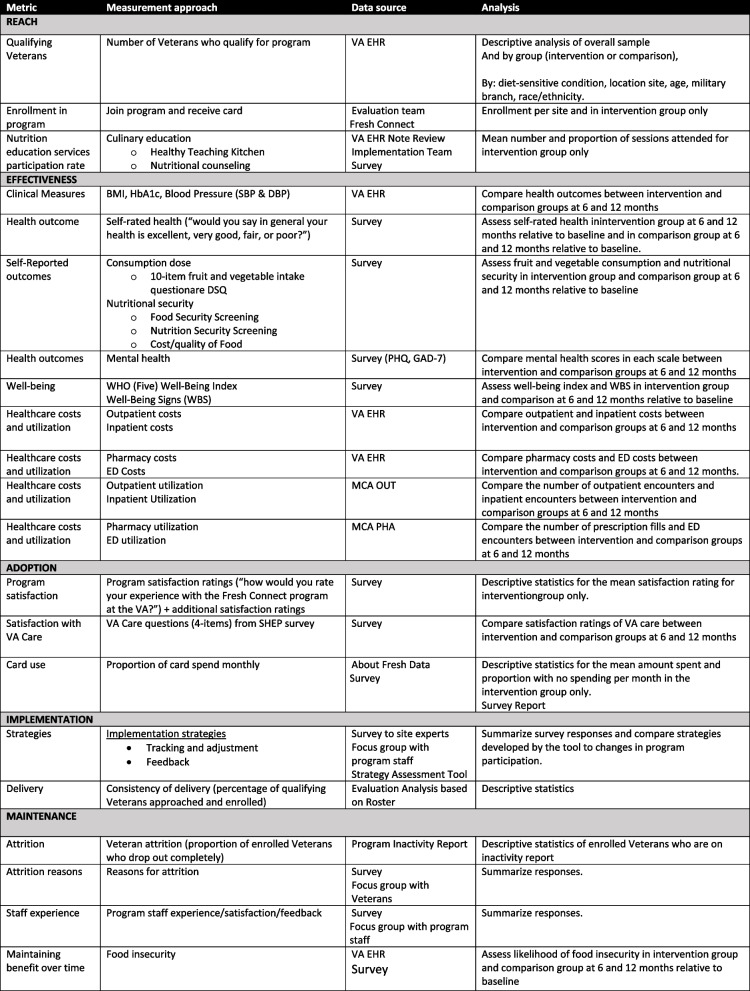
Metrics Overview Based on PRISM-REAIM

Program enrollees and the subsample of comparison group Veterans are invited to complete a survey three times during the study: at baseline shortly after enrollment, 6 months after enrollment, and 12 months after enrollment. Potential survey participants (anticipated *n* = 1000) are mailed an opt-out letter for the survey. If they do not opt out within 3 weeks, they are sent a survey to complete using the online platform Qualtrics, with the first page containing a consent document (survey completion implies consent) [45]. Each survey includes structured measures including the food security screening questions used in VA care known as the Hunger Vital Sign™ [46], a nutrition measure including reports of fruit and vegetable intake (National Cancer Institute Dietary Screener Questionnaire), [47], a validated single-item self-reported health scale [48], and assessments of anxiety and depression, among other measures [49] (see Table 3 for complete list of survey measures). After completion of each individual survey, enrollees will receive a $10 gift card. After completing all three surveys, enrollees will have received a total of $30 in gift cards.

**Table 3:** Survey Components and Time Points

Concept	Measurement Tool	Group	Time Point
Food Security	Household Food Security questionnaire [50, 51]	Tx and Comparison	1, 2, 3
Food Security Screening	Office of Food Security VA Food Insecurity Self Screening questionnaire [52]	Tx and Comparison	1, 2, 3
Nutrition Security Screening	2-item Nutrition Security Screener (NSS) [53]	Tx and Comparison	1, 2, 3
Nutrition Insecurity	Modified: National Cancer Institute’s Dietary Screener Questionnaire [47]	Tx and Comparison	1, 2, 3
Self-rated Health	Self-health-related quality of life Survey [48]	Tx and Comparison	1, 2, 3
Food Cost	Cost and Quality Questionnaire [54]	Tx and Comparison	1, 2, 3
Well-Being	Well-Being Signs Measurement [55]	Tx and Comparison	1, 2, 3
Well-Being	The World Health Organization- Five Well-Being Index (WHO-5) [56]	Tx and Comparison	1, 2, 3
Depression	Patient Health Questionnaire [49]	Tx	1, 2, 3
Anxiety	Generalized Anxiety Disorder Screen (GAD-7) [57]	Tx	1, 2, 3
Benefit Usage	About Fresh Card Usage Questionnaire	Tx	1, 2, 3
Patient Satisfaction with VA Care	Experience of Care Measures (VA Survey of Healthcare Experiences of Patients (SHEP) [58]	Tx and Comparison	1, 2, 3
VA FCPRx Satisfaction	Program Satisfaction Questionnaire [59]	Tx	1, 2, 3
Program Recommendation	Net Promoter Score [60]	Tx	1, 2, 3

### Data management

EHR-based demographic, health, and healthcare utilization data are stored within the VA Informatics Network Computing Infrastructure (VINCI), a secure VA computing environment. [61] Survey data from the Qualtrics survey are stored on secure University of Utah servers. Data relating to produce prescription card usage (allocated funds used per month, shopping trips per month, store locations) are transferred from Fresh Connect to the study team via a data transfer portal. Data management teams screen data for missingness and coherence of survey responses.

Three strategies will be employed to manage missing data. (1) A multiple imputation approach [62, 63] will involve creating multiple plausible imputations for missing covariate values, to maintain statistical power and reduce bias. (2) Inverse Probability Weighting [64, 65] will be used to adjust for the potential biases associated with non-random missingness in the outcome. (3) A missing indicator method [66, 67], where a binary indicator for missingness was included in the analyses, to directly account for the impact of missing categorical data on our study outcomes. These combined approaches will allow us to address different aspects of the missing data, ensuring a comprehensive analysis of the results.

### Outcome variables

#### Outcomes

Our primary outcomes will be clinical measures captured in the VA electronic health record, including BMI (constructed from height and weight values) and blood pressure readings (systolic and diastolic), which will be obtained from the Vital Status table in the VA Corporate Data Warehouse (CDW). In addition, HbA1c will be obtained from CDW laboratory data. A power analysis revealed that our planned sample will result in minimal detectable differences for HbA1c of 0.6 mmol/mol, for systolic blood pressure of 6.5 mm and 3.5 Hg for diastolic blood pressure, and 2.4 in BMI units (weight pounds x height inches) × 703).

We will construct healthcare cost variables from VA Managerial Cost Accounting datasets, which is an activity-based cost accounting system that maps VHA resource expenditures to individual patient encounters. Healthcare costs will be captured separately for outpatient, inpatient, emergency department, and pharmacy services. We will sum these costs for healthcare encounters occurring over the follow-up period and adjust for inflation to 2025 US dollars. Similarly, healthcare utilization will be calculated as the number of outpatient and emergency department visits, prescriptions, and inpatient days. These will serve as important secondary outcomes.

#### Implementation metrics

Implementation processes include an interview approach based on the RE-AIM framework to assess implementation processes and experiences by site to understand adaptation. We will also assess implementation strategies by recording field notes and conducting brief inquiries with implementation staff. Implementation outcomes are measured by characteristics, implementation program data (e.g., site capacity by FTE for staff, and number of nutritionists and dietitians per qualifying Veteran by site).

#### Independent variables

To reduce the influence of confounding variables on our estimates, we will consider a number of measurable patient characteristics in our analyses. Demographic characteristics will include age, race, gender, marital status, priority group, insurance status, and rurality. Clinical characteristics will include the Charlson comorbidity index and the presence of mental health diagnoses. Additional information about clinical metrics and additional measures is shown in Table 2.

### Data analysis

At baseline, descriptive analyses of participants and the comparison group will assess any group differences in their characteristics (implementation or comparison), diet-sensitive condition, location site, age, military branch, and race/ethnicity. The index date for the treatment group will be the date of enrollment in the VA FCPRx program, while the index date for the comparison group will be the first date during our enrollment window in which the Veteran was listed on the eligibility roster and screened positive for food insecurity. We will construct propensity scores for program enrollment using multivariable logistic models including the independent variables listed above and use these propensity scores to conduct inverse probability treatment weighting (IPTW) analyses to compare outcomes between individuals in the FCPRx group and the comparison group. For each of these variables, our primary measure will be the value most proximal within a 2-month window surrounding the follow-up time period (i.e., 6 and 12 months). In secondary analyses, we will calculate averages for individuals with multiple values. In addition, our primary analyses will carry forward baseline values for individuals with missing values in the follow-up period. In exploratory analyses, we will include only those with non-missing outcome values.

In our primary analysis, we will assess differences in clinical measures, including body mass index (BMI), systolic blood pressure (SBP), diastolic BP (DBP), A1c, and self-reported health between patients in the treatment and comparison groups. For each outcome of interest, we will use a linear model with an ITPW approach to estimate the treatment effect and ensure the balance of baseline covariates, including baseline clinical measures. As a secondary analysis, we will compare within-person changes in clinical measures between the two groups.

Secondary outcomes include healthcare cost and utilization, and self-rated health. Exploratory outcomes include fruit and vegetable consumption, nutrition security, depression symptoms, and well-being indices. We will assess changes in secondary and exploratory outcome measures for those participants with both baseline and follow-up values. We will use generalized linear models and two-part models for healthcare cost outcomes and Poisson or negative binomial models for healthcare utilization outcomes. All comparisons will be performed at both 6- and 12-month follow-up intervals.

We will use an approach in which we will assign participants to the VA FCPRx group if they enrolled in the VA FCPRx program regardless of whether or how much of the monetary and nutrition education benefit they used over the 12-month follow-up period. In exploratory analyses, we will use a sub-analysis approach to compare enrolled patients by outcomes based on the amount of financial and educational benefit used.

Our primary analyses will consider all enrolled patients in the VA PRx group. In subgroup analyses, we will break the VA PRx patients into mutually exclusive categories based on the amount of produce purchased and adherence to the educational component of the program. For financial benefit, we will compare participants who used their card minimally, moderately, and fully. These categories will be derived based on the distribution of card use in the sample. For the educational benefit, we will compare participants who did not attend culinary education classes at all, to those who participated some of the time, to those who participated in all 4 classes. We will assess comparison group participants by reasons for non-enrollment, including not being approached for enrollment or declining to enroll. Results of these sub-analyses will inform discussions of potential selection biases and the impact on results. We may perform additional subgroup analysis, including by VHA site and other demographic differences such as race or ethnicity. Qualitative data (e.g., interview transcripts) will be analyzed using approaches including thematic analysis by an experienced qualitative team [68].

## Discussion

There is a growing emphasis on incorporating screenings related to social determinants of health and providing treatment and support within healthcare settings [69]. Despite this, few studies to date incorporate food security screening in health settings, produce prescription interventions, and examination of healthcare outcomes using EHR data. VA FCPRx will produce important evidence about the effectiveness of produce prescription programs for Veterans within an integrated healthcare system. Strengths of this study include the relatively large size of the sample and 12-month duration of the produce prescription program. Many programs do not last more than 6 months. In addition, VA FCPRx provides access to produce in grocery stores, which are generally more accessible than other options [70].

VA FCPRx incorporates many factors crucial to Food is Medicine intervention design [23, 71]. Specifically, health system resources, integration of program activities (e.g., food security screening) into the EHR [72], and design based on clinical logistics that allow for program sustainment are all incorporated [73]. The United States Department of Agriculture (USDA) Gus Schumacher Nutrition Incentive Program (GusNIP) Training, Technical Assistance, Evaluation, and Information Center (NTAE) center recommends that all food prescription evaluations include survey measures of self-rated health (a predictive health outcome) and program satisfaction, which are included in the VA FCPRx protocol.

VA FCPRx is implemented using a clinically embedded process from enrollment to outcomes assessment. Pragmatic designs have unique potential because they are highly scalable in complex health organizations, particularly when integrated with the EHR [74]. EHR processes are embedded within multiple aspects of VA FCPRx, which can be adopted at other VA sites and by other healthcare systems [75]. VA FCPRx is supported by a large, interdisciplinary team of researchers, social workers, dietitians, educators, administrators, community partners, and government leaders. The team worked together closely throughout the development of the protocol. The study is thus designed by multiple partners with a strong understanding of administrative, clinical, and research challenges that may arise during a study of this type.

Implementation of clinically-based food security programs in other healthcare systems has noted challenges with health system resources and in particular with integration of program activities (e.g., food security screening) into the EHR [72]. However, VA FCPRx is conducted in a healthcare system that has incorporated food security screening successfully for over 6 years [46]. A qualitative study examined implementation in 5 different clinic-based produce prescription programs in the U.S. with clinic staff interviews [73]. Adoption of the studied produce prescription programs was generally enthusiastic, implementation was primarily focused on logistics (e.g., identification of patients who met study criteria for enrollment, distribution and tracking of incentives), and sustainment was a concern [73].

### Limitations

It is important to acknowledge the limitations of VA FCPRx. Because eligibility screening data are EHR-based, we will not have results from Veterans who do not use VA care regularly. The comparison group will meet eligibility criteria but did not enroll in the program for a variety of reasons. This design choice was made to accommodate the clinical embedding approach and to offer the intervention to as many eligible Veterans experiencing food insecurity as possible during an enrollment period. However, because of the inherent differences in patients who do and do not enroll in the program, our estimated effects of the program on patient outcomes will be subject to confounding bias. Our IPTW approach should minimize the impact of measured confounders, but unmeasured confounders may still be present. Therefore, other designs (e.g., cluster randomization) may be important to examine in future work. There may be a high non-response rate for the survey, particularly in the comparison group, and all analyses will report the response rate and details of enrollment procedures. We will report characteristics of both study sites, including the number of VA nutritionists and social workers supported by the site, and we know that participating sites do not have parallel levels of support. Culinary education is offered as part of VA programming and there were no study resources assigned to this programming which may impact the effectiveness of this support. Furthermore, comparison group participants are eligible for this VA benefit and may also participate in culinary education if interested.

## Conclusions

If VA FCPRx is found to be effective, there is strong potential to scale up this program and support successful, evidence-based implementation throughout the VA healthcare system. In addition, many of the approaches employed could be adapted for other healthcare systems. This program has the capacity to improve health and healthcare for a population, Veterans, a population who have worse health outcomes.

## Supplementary Information

Additional file 1. EHR-integrated template.

## Data Availability

The data that support the findings of this study may be available from VHA but restrictions apply to the availability of these data, which were used under appropriate approvals, authorizations, and in accord with VHA and University of Utah policy and so are not publicly available. Data are however available from the authors upon reasonable request and with permission of VHA and an appropriate Memorandum of Understanding and permissions.
