# Supplementary material for: Veterans Affairs FreshConnectProduceRx: a study protocol for a pragmatic quasi-experimental study assessing health, healthcare costs, and implementation processes of a produce prescription program in VA medical centers
Source: BMC Public Health. 2025 Jul 3;25:2353. doi: 10.1186/s12889-025-23355-2 (PMC12225373; doi:10.1186/s12889-025-23355-2)
Supplement: Supplementary file 1 — Additional file 1. EHR-integrated template. [file 12889_2025_23355_MOESM1_ESM.pdf]

Step 1: Veteran screens positive for food insecurity

The Veteran reports the following (Food Insecurity screening)

1. Within the past 12 months, you worried whether your food would run out before you got money to buy more.
   a. Answer selection (Often/Sometimes/Never true)

2. Within the past 12 months, the food you bought just didn't last and you didn't have money to get more.

   a. Answer Selection (Often/Sometimes/Never true)

Step 2: Veteran agrees to referral to Dietitian/Social work/or already receiving services.

****** This patient has screened positive for Food Insecurity and may be eligible to participate in a Produce Prescription Pilot Study. ******

PRODUCE RX FRESH CONNECT PROGRAM INTAKE NOTE FOR CLINICIAN COMPLETING THE NOTE: The Pilot Program includes a pre-paid Master Card of $100/month for up to 12 months to purchase fresh fruits and vegetables at participating stores. Participating stores in VHASLCHCS include Walmart, Kroger (Smith's, Fred Meyer), Albertson’s and Lucky. This program also includes attendance at a virtual 4-week Nutrition Healthy Teaching Kitchen group class that will include cooking demonstrations on how to prepare fruits and vegetables. Alternatively, patients may be able to participate in individual nutrition counseling with a dietitian. Check this link to see if patient is eligible for Produce Rx program based on financial criteria (Veteran priority group 5 or 7) and nutrition sensitive conditions (dx DM, HTN, or BMI >/= 30):

Fresh Connect Produce Rx Secure Study Eligibility

Step 3: confirmation of meeting criteria

1. Author confirms patient meets the criteria for participation in Produce Rx Fresh Connect Program.

2. Patient is on the roster.

Step 4: PROVIDE PROGRAM INFORMATION TO THE VETERAN:

The VHASLCHCS has a program that provides $100/month

on a pre-paid Master Card for up to 12 months to buy fresh fruits and vegetables at participating stores.

Participating stores in the VHASLCHCS include Walmart, Kroger (Smith's, Fred Meyer's), Albertson’s and Lucky. The program also includes attendance at a virtual 4-week Nutrition Healthy Teaching Kitchen group class that will include cooking demos on how to prepare fruits and vegetables. Alternatively, patients can participate in individual nutrition counseling with a dietitian.

Step 5: questions for veterans:

1. Is the patient willing to shop, prepare and eat the produce? (Yes)
2. Does the patient have transportation to participating stores? (Yes)
3. Is the patient willing and able to attend a virtual 4-week healthy teaching kitchen (HTK)cooking class program? This is the most complete nutrition learning experience for this program. The virtual HTK class is held on Thursdays from 2-3:30pm. (Yes, Veteran is willing to see a Dietitian for individual nutrition counseling)

Step 6: Release of Information (ROI) link VA-FORM 10-5345 for authorization to release Personally Identifiable Information to Fresh Connect. This form is MANDATORY to enroll Veterans in the Fresh Connect platform and receive the debit card.

* Clinician will send form and patient agreed to sign and return form via Mail (return envelope provided).

Step 7: Fresh Connect Enrollment handout given and reviewed with patient.

Fresh Connect Produce Rx Enrollment Handout: For the VA to know if this program is beneficial for participating Veterans, Veterans will receive 3 surveys, including one at the start of the program, one at 6 months, and one at 12 months throughout the program. This will help the VA demonstrate how this program impacts Veterans and may help other Veterans get access to the program. We appreciate your help in completing the surveys.

Step 8: Read Fresh Connect disclosure to Veteran:

"Information shared and/or collected by About Fresh, including your use of Fresh Connect card, your purchases with the Fresh Connect Card, and

self-reported information, may be shared with the VA Salt Lake City Health Care System and University of Utah evaluation team."

Step 9: VERIFY CONTACT INFORMATION WITH VETERAN

Veterans will not receive the debit card without correct contact/mailing information.

- Cardholder info:
- Name:
- DOB:
- Phone:
- Cell Phone:
- Preferred Language: English Email:
- Address:
- Corrections to contact/mailing information if any:
- Preferred method of contact (check all that apply): Phone/Text Message/Email
- Number of people in household (including cardholder):
- Number of people in household under the age of 18:

Step 10: Please read the following questions out loud:

1. In the last 30 days, have you or anyone in your household received SNAP benefits or food stamps? (No/Yes)
2. In the last 30 days, have you or anyone in your household received WIC (Women, Infants, and Children) benefits? (No/Yes)
3. Does anyone in your household participate in Medicare? (No/Yes)
4. Does anyone in your household participate in Medicaid? (No/Yes)
5. Would you say that in general your health is excellent, very good, fair, or poor?

THE ROI MUST BE SIGNED AND SENT TO CONTACT IN QUESTION D ABOVE. VETERAN WILL NOT BE ABLE TO PARTICIPATE IN THE FRESH CONNECT PROGRAM WITHOUT IT.
